# The Dyslexia-susceptibility Protein KIAA0319 Inhibits Axon Growth Through Smad2 Signaling

**DOI:** 10.1093/cercor/bhx023

**Published:** 2017-02-17

**Authors:** Filipa Franquinho, Joana Nogueira-Rodrigues, Joana M. Duarte, Sofia S. Esteves, Christin Carter-Su, Anthony P. Monaco, Zoltán Molnár, Antonio Velayos-Baeza, Pedro Brites, Mónica M. Sousa

**Affiliations:** 1 Nerve Regeneration group, Instituto de Biologia Molecular e Celular – IBMC and Instituto de Inovação e Investigação em Saúde, University of Porto, 4200-135 Porto, Portugal; 2 Instituto de Ciências Biomédicas Abel Salazar – ICBAS, 4050-313 Porto, Portugal; 3 Department of Molecular and Integrative Physiology, University of Michigan Medical School, Ann Arbor, MI 48109-22, USA; 4 The Wellcome Trust Centre for Human Genetics, Oxford OX3 7BN, UK; 5 Office of the President, Ballou Hall, Tufts University, Medford, MA 02155, USA; 6 Department of Physiology, Anatomy, and Genetics, University of Oxford, Oxford OX1 3QX, UK

**Keywords:** axon growth, axon regeneration, dyslexia, KIAA0319, Smad2

## Abstract

KIAA0319 is a transmembrane protein associated with dyslexia with a presumed role in neuronal migration. Here we show that KIAA0319 expression is not restricted to the brain but also occurs in sensory and spinal cord neurons, increasing from early postnatal stages to adulthood and being downregulated by injury. This suggested that KIAA0319 participates in functions unrelated to neuronal migration. Supporting this hypothesis, overexpression of KIAA0319 repressed axon growth in hippocampal and dorsal root ganglia neurons; the intracellular domain of KIAA0319 was sufficient to elicit this effect. A similar inhibitory effect was observed in vivo as axon regeneration was impaired after transduction of sensory neurons with *KIAA0319*. Conversely, the deletion of *Kiaa0319* in neurons increased neurite outgrowth in vitro and improved axon regeneration in vivo. At the mechanistic level, KIAA0319 engaged the JAK2-SH2B1 pathway to activate Smad2, which played a central role in KIAA0319-mediated repression of axon growth. In summary, we establish KIAA0319 as a novel player in axon growth and regeneration with the ability to repress the intrinsic growth potential of axons. This study describes a novel regulatory mechanism operating during peripheral nervous system and central nervous system axon growth, and offers novel targets for the development of effective therapies to promote axon regeneration.

## Introduction

In the developing brain, correct axon growth and pathfinding are essential for accurate circuit formation and processing of information. In fact, several neurodevelopmental disorders are known to be related to defects in axon growth and circuitry, including schizophrenia (Muraki and Tanigaki 2015), epilepsy (Thom et al. 2004), autism spectrum disorders (Reiner et al. 2016), and dyslexia (Linkersdorfer et al. 2012). Following the establishment of connections, the axon growth capacity severely declines. As a consequence, in the adult central nervous system, following either injury or disease, axon (re)growth and guidance generally fail. The cell intrinsic mechanisms that regulate axon growth during development and axon regeneration in the adult remain poorly understood.

**Figure 1.**
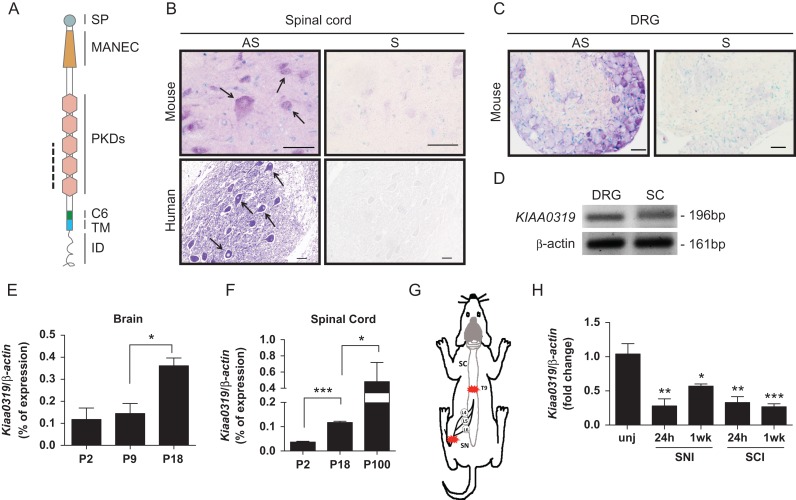
Expression of Kiaa0319 in nervous tissue during development and after injury. (*A*) Schematic representation of KIAA0319 domains: SP, signal peptide; MANEC, motif at N-terminus with 8 cysteines; PKD; polycystic kidney disease; C6, domain with 6 cysteines; TM, transmembrane; ID, intracellular domain. The dashed line indicates the region corresponding to the in situ hybridization probe. (*B*) Representative microphotographs of in situ hybridization of *Kiaa0319/KIAA0319* in ventral horn spinal cord neurons from mice (upper, P60, *n =* 4; scale bar, 20 μm) and human (lower, 33 years-old, *n =* 1; scale bar, 50 μm). AS, antisense probe; S, sense probe. Arrows in *B* highlight labeled neurons. (*C*) In situ hybridization of *Kiaa0319* in mouse DRG (P20, *n =* 3). AS, antisense probe; S, sense probe; scale bar, 50 μm. (*D*) RT-PCR of *KIAA*0319 and β-actin (loading control) in human DRG and human spinal cord (SC) samples. (*E, F*) Quantitative-PCR analysis of the levels of *Kiaa0319* expression in the mouse brain (*E*) and spinal cord (*F*) at postnatal days 2, 9, 18, and 100 (*n =* 4 each sample/age). (*G*) Schematic representation of the lesion model used to assess *Kiaa0319* expression. L4, L5, and L6 DRGs were collected 24 h or 1 week after sciatic nerve (SN) or spinal cord (SC) transection at the T9 level. Red stars indicate lesion site. (*H*) *Kiaa0319* mRNA expression levels 24 h and 1 week after either sciatic nerve (SNI) or spinal cord transection (SCI); unj = uninjured. Results were compared with the uninjured condition and are expressed as mean ± SEM. *P < 0.05, **P < 0.01, ***P < 0.001.

In a transcriptomic-based analysis using Affymetrix microarrays (GeneChip Mouse Genome 430 2.0 Arrays), our group identified *Kiaa0319* as a highly expressed gene in adult dorsal root ganglia (DRG) neurons (unpublished data). The human *KIAA0319* gene has several splicing variants (Velayos-Baeza et al. 2007) although the predominant form is the full-length transcript encoding isoform A of the protein with 1052 amino acids and 116 kDa that can assemble into a highly glycosylated dimer (Velayos-Baeza et al. 2008). KIAA0319 is very conserved among species. It contains a single transmembrane (TM) domain with a cytoplasmic C-terminus, and in the extracellular region 5 polycystic kidney disease (PKD) domains, and 2 cysteine-rich motifs: a motif at N-terminus with eight cysteines (MANEC) and a motif with 6 cysteines (C6) just before the TM domain (Velayos-Baeza et al. 2007) (Fig. 1*A*). Given the importance of PKD domains in cell–cell/cell–matrix interactions (Ibraghimov-Beskrovnaya et al. 2000), a putative role of KIAA0319 during neuronal migration was suggested. The MANEC domain of KIAA0319 is highly similar to the PAN/apple domain described as a mediator of protein–protein interactions (Hong et al. 2015). Interestingly, *KIAA0319* is located in the dyslexia susceptibility *locus DYX2* (Dyslexia Susceptibility 2) that resides on chromosome 6p22 being the most consistently replicated in this disorder (reviewed in Carrion-Castillo et al. 2013). KIAA0319 was found to be expressed less in individuals with a specific haplotype associated with dyslexia—variant rs9461045 (Paracchini et al. 2006), later on proven to be caused by the introduction of a binding site for the transcription factor OCT1 in the *KIAA0319* promoter (Dennis et al. 2009). Decreasing specifically the expression of KIAA0319 by in utero electroporation of short-hairpin RNA (shRNA) in rats was reported to impair cortical neuronal migration, which could be rescued by overexpressing the full-length gene (Paracchini et al. 2006; Peschansky et al. 2010). This suggested that KIAA0319 participates on radial migration in the developing rat neocortex (Paracchini et al. 2006). However, a recent study on *Kiaa0319* knock-out mice indicates that lack of KIAA0319 does not have any obvious effect on mouse brain development suggesting that, at least in mice, this protein is not required for neuronal migration (Martinez-Garay et al. 2016).

Besides being expressed in different regions of the developing mouse and human brain (Paracchini et al. 2006), our transcriptonic analysis and data available in the Allen brain atlas (http://mousespinal.brain-map.org) support that KIAA0319 is also highly expressed in spinal cord neurons and in DRG neurons of juvenile and adult animals, thus further suggesting that this protein has additional roles unrelated to its putative involvement in cortical neuronal migration during development. The current study is aimed at clarifying the biological role of KIAA0319 in neurons. Our results provide evidence that the KIAA0319 cytosolic domain modulates axon growth and regeneration through Smad2 activation.

## Materials and Methods

### Animals

NMRI mice and Wistar rats (8- to 10-week-old) of either sex were handled according to European Union and National rules, maintained under a 12-h light/dark cycle and fed with regular rodent chow and water ad libitum. In all animal experiments, the investigator was blinded to the group allocation. C57BL/6J-*D130043K22Rik*^*tm1c(KOMP)Wtsi*^ mice carrying an exon 6 floxed *Kiaa0319* allele (*Kiaa0319-Flx; Kiaa0319*^*F/F*^) were generated at the Wellcome Trust Centre for Human Genetics (Oxford, UK) using “knockout-first”-targeted stem cells (Skarnes et al. 2011) from the Knock-Out Mouse Project (KOMP) repository at UC Davis, CA (www.komp.org) and have been described elsewhere (Martinez-Garay et al. 2016). To generate a neuron-specific *Kiaa0319* deletion in adult animals, *Kiaa0319*^F/F^ mice were crossed with Slick-H mice (from Dr Guoping Feng, Duke University Medical Center) that co-express inducible-CreER^T2^ and yellow fluorescent protein (YFP) under the control of the neuronal *Thy1* promoter (Young et al. 2008). The resulting *Thy1-cre*^*+*^*Kiaa0319*^*F/+*^ mice were selected and crossed with *Kiaa0319*^*F/+*^ mice so that *Thy1-cre*^*+*^*Kiaa0319*^*F/F*^ mice were generated. Recombination was induced by injecting i.p. 20 mg/mL of tamoxifen for 5 days starting at postnatal day 21 (P21). Cre-mediated deletion of exon 6 generates a predicted p.D374VfsX14 effect at the protein level, leading to the absence of Kiaa0319. Given the neuroprotective effects of tamoxifen (Wakade et al. 2008), tamoxifen-treated *Thy1-cre*^*+*^*Kiaa0319*^*+/+*^ littermates were used as controls.

### Human Samples

Adult human spinal cord and DRG were obtained from the NICHD Brain and Tissue Bank for Developmental Disorders at the University of Maryland, Baltimore, MD. Both 10% formalin-fixed spinal cord, and frozen DRG and spinal cord samples were derived from healthy adult males.

### **In Situ** **Hybridization**

Four micrometer sections of human and mouse spinal cord and DRG were incubated with 50 µg/mL proteinase K, refixed in 10% formalin and treated with 0.5% acetic anhydride. RNA probes for human *KIAA*0319 and for its mouse homolog *D130043K22Rik* (NM_001081051) (both antisense and control sense probes) were synthesized and labeled with digoxigenin using the DIG RNA Labeling Kit (SP6/T7) (Roche). After incubating sections with prehybridization solution (50% formamide, 4×  SSC) for 2 h at 37°C, denatured probes (0.5 µg/µL) were mixed in hybridization buffer (40% formamide, 4× SSC, 1 mg/mL denatured sheared salmon sperm DNA, 1 mg/mL yeast t-RNA, 10 mM DTT, 10% dextran sulfate, 1× Denhardt's reagent) overnight. The stringency of washes varied according to the probe used. Following incubation for 2 h (37°C) with sheep anti-DIG-alkaline phosphatase antibody (1:1000, Roche), sections were developed overnight using NBT/BCIP (Roche). Photos were taken using an Olympus DP 25 microscope.

### RNA Extraction, Reverse Transcription PCR, and Quantitative Real-time PCR

RNA from mouse DRG, spinal cord, and brain at postnatal day 2, 18, and 100 (P2, P18, and P100) (*n =* 4 of each age) and adult human DRG and spinal cord (NICHD Brain and Tissue Bank for Developmental Disorders at the University of Maryland, Baltimore, MD) was extracted using NZY Total RNA Isolation Kit (NZY Tech). Mouse DRGs (L4–L6) were collected 24 h and 1 week after either sciatic nerve or spinal cord transection (*n =* 5 each). RNA concentration and purity were determined by NanoDrop® spectrophotometry, and integrity was assessed using BioRad's Experion RNA chip. cDNA synthesis was performed with SuperScript™ First-Strand Synthesis System for RT-PCR (Invitrogen). Reverse transcription PCR (RT-PCR) for human samples was performed using specific primers for *KIAA0319* (5’-TGGACTCGGACATTAAGG-3’, in exon 16, forward, and 5’-CAACCTGCTGTATCAACC-3’, in exon 17, reverse, for amplification of a 196 bp fragment) and β-actin (5’-ACCACACCTTCTACAATGAG-3’, forward, and 5’-TAGCACAGCCTGGATAGC-3’, reverse). Quantitative real-time PCR (qRT-PCR) for mouse samples was done with the iQ™ SybrGreen Supermix Kit (Biorad) using specific primers designed with Biorad Beacon designer software for β-actin (5’-TATGCTCTCCCTCACGCCATCC-3’, forward, and 5’-TGTCACGCACGATTTCCCTCTC-3’, reverse) and *Kiaa0319* (5’-CCAGTAGACTTCCAAGGT-3’, in exon 6, forward, and 5’-CCACTCTGAAGGCATAGA-3’, in exon 7, reverse, for amplification of a 91 bp fragment). All samples were run in triplicate, qPCR analysis was performed with BioRad's iQ5 2.1 software and quantification was done using the 2^−ΔΔCt^ (Livak) method (Livak and Schmittgen 2001).

### Expression Constructs

Full-length and different deletion human *KIAA0319* cDNA constructs have been previously described (Velayos-Baeza et al. 2008; Levecque et al. 2009; Velayos-Baeza et al. 2010). Inserts of these plasmids were sub-cloned into the pCAGIG vector to obtain the expression constructs used in this work (Table 1). Multiple alignment of mammalian KIAA0319 (region encoded by exons 19–21, containing the TM and cytosolic domains) was performed using Clustal Omega (http://www.ebi.ac.uk/Tools/msa/clustalo/). Putative phosphorylation sites within the KIAA0319 cytosolic tail were predicted using PhosphoNet (http://www.phosphonet.ca, Kinexus Bioinformatics Corporation). Single missense mutants of predicted phosphorylation sites between positions 984 and 1023, corresponding to the juxtamembrane cytosolic fragment deleted in construct hKAd20-21a, were obtained from the human KIAA0319 full-length construct (hKA) (cloned in pcDNA4) using the QuikChange® kit (Stratagene) and 2 mismatched primers introducing one or 2 bp substitutions in the original sequence. To generate the deletion mutant where the highly conserved 1024-SSLMVSE-1030 intracellular region was replaced by 2 glycine residues, the following pair of primers was used: 5’-GGGTCTGAGTTTGACAGTGACCAGGACACAATCTTC-3’, forward, and 5’-CCCGTTGTGCTCTGTGCTTCGGTGCTT G-3’, reverse. Modified plasmid inserts were confirmed by DNA sequencing.

**Table 1:** Constructs for expression of human KIAA0319 used in this work

Plasmid^a^	Protein^b^	Del aa^c^	New^d^	No. aa	Tagging aa^e^	Size^f^	Comments
pCAGIG	Control						Empty vector (with IRES-eGFP)
pCAGIG-hKA	hKA	—	—	1072	—	117.76	Full-length
pCAGIG-hKAd3a	hKAd3a	23–134	—	960	—	105.18	Deletion of MANEC domain
pCAGIG-hKAd5-15	hKAd5-15	345–812	T	605	—	67.19	Deletion of PKD domains
pCAGIG-hKHAmd3-18	hKAd3-18	23–952	*RPED	183	156–183	21.15	Deletion of MANEC-to-C6 domains
pCAGIG-hKAmd20-21	hKAd20-21	984–1072	—	1009	984–1009	110.37	Deletion of whole cytoplasmic domain
pCAGIG-hKAmd20-21a	hKAd20-21a	984–1023	—	1060	1033–60	116.11	Deletion of juxtamembrane region of cytoplasmic domain
pCAGIG-hKAmd21a	hKAd21b	1031–72	—	1058	1031–58	116.33	Deletion of C-terminal region of cytoplasmic domain
pCAGGS-hKAGd5-15	hKAGd5-15	345–812	T	866	606–866	96.57	Deletion of PKD domains, with C-terminal eGFP tag
pcD4KA	KIAA0319	—	—	1072	—	117.76	Full-length
pcD4KA-T993A	hKA-T993A	993	A	1072	—	117.72	Full-length, T993A mutant
pcD4KA-Y995A	hKA-Y995A	995	A	1072	—	117.66	Full-length, Y995A mutant
pcD4KA-Y1013A	hKA-Y1013A	1013	A	1072	—	117.66	Full-length, Y1013A mutant
pcD4KA-S1019A	hKA-S1019A	1019	A	1072	—	117.74	Full-length, S1019A mutant
pcD4KAdSSLMVSE	hKAdSSLMVSE	1024–30	GG	1067	—	117.08	Short deletion in cytoplasmic domain

^a^Plasmid names show the vector backbone; pcD4 = pcDNA4-TO.

^b^Protein name used in the text; hKA: human KIAA0319.

^c^Residues deleted from the full-length protein.

^d^New residues introduced in place of the deleted protein fragment, excluding any C-terminal tagging sequence. For hKAd3-18, this sequence includes an N-terminal HA (*bold*) tag (**YPYDVPDYA**RPED).

^e^Residues derived from the vector sequence including the C-terminal tag. All of them have the myc (*bold*) + His tag ([L/I/F][E]SRGPF**EQKLISEEDL**NMHTGHHHHHH) except hKAGd5-15 (22 aa + eGFP).

^f^Size (kDa) of the deduced protein, including the SP (20 aa /1.94 kDa, removed in final protein).

### DRG Neuron Cultures

DRG neuron cultures were performed as detailed in Miranda et al. (2011) from 8-week-old Wistar rats or from *Thy1-cre*^*+*^*Kiaa0319*^*F/F*^ and *Thy1-cre*^*+*^*Kiaa0319*^*+/+*^ mice. In the case of DRG from Wistar rats, *KIAA0319* was overexpressed in an IRES-eGFP backbone (plasmid pCAGIG-hKA; Table 1) by nucleofection (4D Nucleofector Amaxa system; CM#138 program). Transfected DRG neurons (200 000 neurons/transfection) were left in suspension for 24 h and thereafter plated in 24-well plate coverslips coated with 20 µg/mL poly-l-lysine and 5 µg/mL laminin. Cells were grown for 12 h in DMEM:F12 supplemented with 1× B27, 1% penicillin/streptomycin, 2 mM l-glutamine and 50 ng/mL NGF. Neurons were fixed with 2% paraformaldehyde and βIII-tubulin immunoreactivity was detected using immunofluorescence (1:2000; Promega). In the case of *Thy1-cre*^*+*^*Kiaa0319*^*F/F*^ and *Thy1-cre*^*+*^*Kiaa0319*^*+/+*^ mice, only YFP-positive neurons were considered. For Wistar rats, only eGFP-positive/βIII-tubulin-positive cells were traced. At least 100 cells per condition were traced. All experiments were repeated at least twice and the investigator was blind to the group allocation. Scholl analysis was performed using Synapse Detector (SynD) software (Schmitz et al. 2011) where the total neurite length and branching and the number of processes crossing concentric circles centered at the cell body, with radiuses of consecutive multiples of 20 μm, were quantified.

### Hippocampal Neuron Cultures

**Figure 2.**
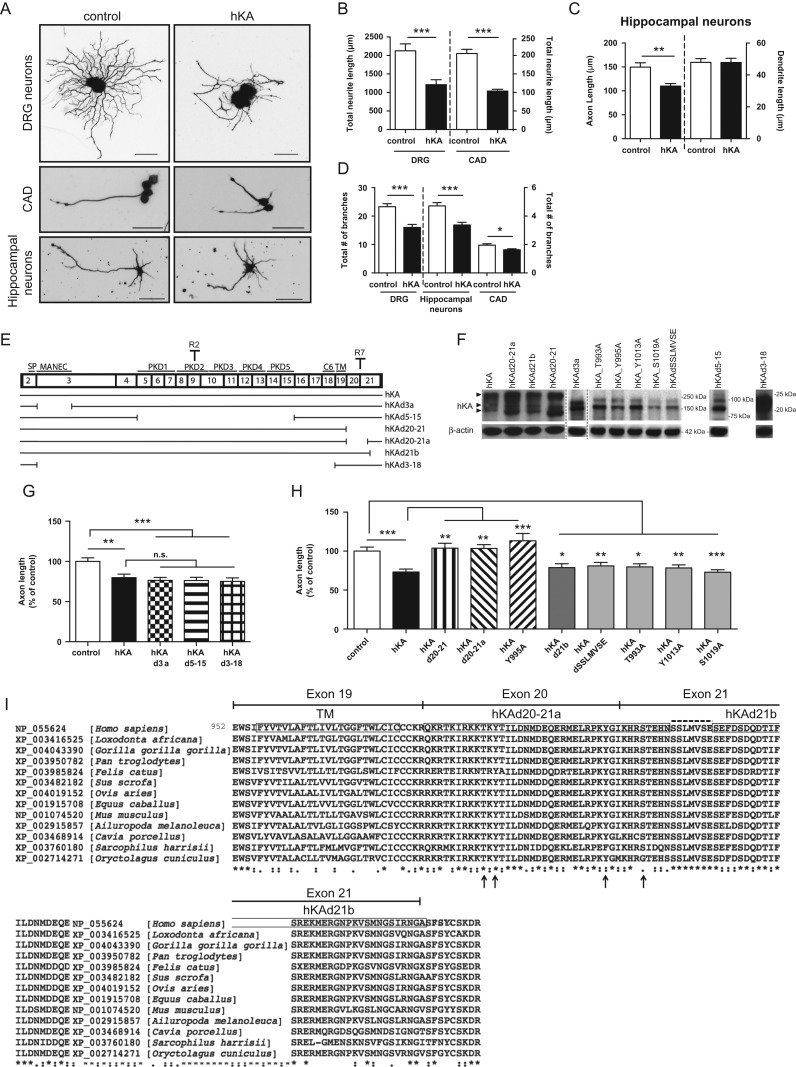
The initial region of the cytosolic domain of KIAA0319 is necessary for inhibition of axon growth. (*A*) Representative anti-βIII-tubulin immunofluorescence images of DRG neurons, differentiated CAD cells and hippocampal neurons transfected with either control empty plasmid or full-length human KIAA0319 (hKA); scale bar, 50 μm. (*B*) Quantification of the total neurite length in transfected DRG (control: *n =* 75; hKA: *n =* 50 neurons) and transfected CAD cells (control: *n =* 162; hKA: *n =* 152 neurons). (*C*) Quantification of axon and dendrite length in transfected primary hippocampal neurons (control: *n =* 105; hKA: *n =* 71 neurons). (*D*) Quantification of the total number of segments in transfected DRG neurons (control: *n =* 68; hKA: *n =* 40), hippocampal neurons (control: *n =* 75; hKA: *n =* 62) and CAD (control: *n =* 162; hKA: *n =* 152 neurons). (*E*) Schematic representation of KIAA0319 constructs (see also Table 1). The numbered upper boxes correspond to the different KIAA0319 exons. In the upper part of the boxes, the region codified is indicated (SP, MANEC domain, PKD domains 1–5, C6 region, and TM domain) together with specific antibody recognition sites for KIAA0319 (R2 in the PKD2 domain and R7 in the cytoplasmic domain). (*F*) Western blot analysis of KIAA0319 (hKA-upper) with R2 antiserum (R7 antiserum for the 2 far right lanes) and β-actin (lower) in CAD cells overexpressing each of the constructs represented in *E*. Arrowheads indicate different protein forms commonly detected after overexpression (Velayos-Baeza et al. 2008): dimers (top), fully glycosylated monomer (middle) and partially glycosylated monomer (bottom). Due to the large deletions in the hKAd3-18 and hKAd5-15 mutants a big shift in molecular weight is observed. Molecular weight markers used in each of the gels are shown. (*G*) Quantification of axon length in hippocampal neurons transfected with control empty plasmid (control; *n =* 105 neurons), full-length KIAA0319 in pCAGIG backbone (hKA; *n =* 71 neurons), hKAd3a (*n =* 66 neurons), hKAd5-15 (*n =* 60 neurons) and hKAd3-18 (*n =* 58 neurons). (*H*) Quantification of axon length in hippocampal neurons transfected with control plasmid (control; *n =* 64 neurons), hKA (*n =* 54 neurons), hKAd20-21 (*n =* 65 neurons), hKAd20-21a (*n =* 62 neurons), hKA Y995A (*n =* 57 neurons), hKAd21b (*n =* 50 neurons), hKAdSSLMVSE (*n =* 74 neurons), hKA T993A (*n =* 58 neurons), hKA Y1013A (*n =* 61 neurons) and hKA S1019A (*n =* 63 neurons). (*I*) Sequence alignment of the TM domain and cytosolic tail of mammalian KIAA0319 proteins (encoded by exons 19–21 of the human gene, as shown on top). The accession number of each species is depicted. Strongly conserved positions are shown below the sequence, as annotated by the alignment software: (*) single, fully conserved residue; (:) residues with strongly similar properties; (.) residues with weakly similar properties. Boxes in the human sequence represent the TM domain and the regions deleted in hKAd20-21a and hKAd21b mutants. The SSLMVSE sequence is highlighted above the alignment with a dashed line. Putative phosphoresidues predicted using PhosphoNET are highlighted with arrows, below the alignment. Results are expressed in mean ± SEM * *P* < 0.05, **P < 0.01, ***P < 0.001; ns, not statistically significant.

Hippocampal neuron cultures were performed as described (Leite et al. 2016) using NMRI E16.5 embryos. Cells were cultured in Neurobasal medium (Invitrogen) supplemented with 1× B27 (Gibco), 1% penicillin/streptomycin (Gibco) and 2 mM l-glutamine (Gibco) on PLL-coated coverslips. Hippocampal neurons (750 000 neurons/transfection) were nucleofected using the 4D Nucleofector Amaxa system (CU#110 program). For the analysis of different KIAA0319 mutants, nucleofection was done with 300 ng of plasmid DNA coding either the full-length or deletion mutants of the KIAA0319 protein (Table 1 and Fig. 2*E*). For neurite tracing, co-nucleofection with 200 ng pmaxGFP™ (Lonza) was done and detection of KIAA0319 was performed using rabbit R2 antiserum (1:500; see Supplementary Fig. 1) (Velayos-Baeza et al. 2008). In the experiments using shRNA for SH2B1 and JAK2, co-nucleofection was performed with KIAA0319 and pmaxGFP™. Analysis of downregulation of expression in hippocampal neurons was done using rabbit anti-SH2B1β (1:2500) (Rui et al. 1997) (see Supplementary Fig. 2) and rabbit anti-JAK2 (Cell Signaling Technology #3230, 1:500) (see Supplementary Fig. 3). Transfected hippocampal neurons were cultured for 96 h. Cells were fixed with 2% PFA. For tracing analyses, immunofluorescence for βIII-tubulin was performed after which axon length of GFP-positive/βIII-tubulin-positive cells was measured using NeuronJ. The axon length, dendrite length, and total number of branches were quantified with ImageJ NeuronJ 1.4.1 plug-in (Meijering et al. 2004). For assays using SM16 (4-(5-(benso[d][1,3]dioxol-5-yl)-4(6methylpyridin-2-yl)1H-imidazol-2-yl)biclyclo[2.2.2]octane-1carboxamide; kindly provided by Dr Engebretsen, Institute for Experimental Medical Research, Oslo University Hospital), 2 μM of drug in DMSO were added before plating, and cells were cultured for 96 h. In experiments using drugs, DMSO was used as control. All experiments were repeated at least twice. In all experiments, the investigator was blinded to the group allocation.

### CAD Cell Cultures

 Cath.-a-differentiated (CAD) cells, derived from a central nervous system (CNS) catecholaminergic cell line (Qi et al. 1997), were cultured in DMEM supplemented with 10% fetal bovine serum (FBS) and 1% penicillin/streptomycin. To induce neurite differentiation, serum was withdrawn for 48 h. For analysis of signaling cascades activated by KIAA0319 overexpression, transfection of the neuronal cell line CAD with either WT KIAA0319 or KIAA0319 deletion mutants was used. Briefly, cells (180 000 cells/well on 24-well plates) were transfected in quadruplicate using Lipofectamine® 2000 (ThermoFisher Scientific) and 500 ng of plasmid DNA. Protein extraction was performed either 24 h or 48 h post-transfection for western blot analysis. For neurite tracing, whenever *KIAA0319* was overexpressed, cotransfection using pmaxGFP™ was performed and KIAA0319 was detected by immunofluorescence using rabbit R2 antiserum (1:500; see Supplementary Fig. 1) (Velayos-Baeza et al. 2008). Sholl analysis was done with SynD software.

### Western Blotting

Protein lysates of spinal cord of adult *Thy1-cre*^*+*^*Kiaa0319*^*F/F*^ and *Thy1-cre*^*+*^*Kiaa0319*^*+/+*^ mice, transfected CAD cells, and hippocampal neurons (25–50 μg/lane) were separated on 12% SDS–PAGE gels, transferred to nitrocellulose, blocked in 5% nonfat dried milk and incubated with the following primary antibodies: rabbit anti-phospho-Smad2 (Ser465/467) (Cell Signaling Technology #3108, 1:1000), rabbit anti-Smad2 (Cell Signaling Technology #5339, 1:1000), rabbit anti-phospho-AKT (Ser473) (Cell Signaling Technology #4060, 1:1000), rabbit anti-phospho-AKT (Thr308) (Cell Signaling Technology #2965, 1:1000), rabbit anti-AKT (Cell Signaling Technology #4961, 1:1000), rabbit anti-phospho-GSK3β (Ser9) (Cell Signaling Technology #9323, 1:1000), rabbit anti-GSK3β (Cell Signaling Technology #9315), rabbit anti-JNK (Cell Signaling Technology #9252, 1:1000), rabbit anti-phospho-ERK (Thr202/Tyr204) (Cell Signaling Technology #4370, 1:2000), rabbit anti-ERK (Cell Signaling Technology #9102, 1:1000), rabbit anti-phospho-Smad1 (Ser463/465)/phospho-Smad5 (Ser463/465)/phospho-Smad8 (Ser465/467) (Cell Signaling Technology #9511, 1:200), rabbit anti-phospho-STAT3 (Tyr705) (Cell Signaling Technology #9145, 1:1000), rabbit anti-STAT3 (Cell Signaling Technology #4904, 1:2000), rabbit anti-JAK2 (Cell Signaling Technology #3230, 1:1000), rabbit anti-SH2B1β (1:50 000) (Rui et al. 1997) and mouse anti-β-actin (Sigma A5441, 1:5000). Overexpressed WT and mutant KIAA0319 proteins were detected using rabbit R2 antiserum (1:5000) (Velayos-Baeza et al. 2008) except for hKAd5-15 and hKAd3-18 where rabbit R7 antiserum (1:5000) was used (Velayos-Baeza et al. 2010) (Fig. 2*E*). In spinal cord lysates R7 was used for detection of KIAA0319. Secondary antibodies were: donkey anti-mouse IgG conjugated with HRP (Jackson Immunoresearch Europe 715-035-151, 1:5000) or donkey anti-rabbit IgG conjugated with HRP (Jackson Immunoresearch Europe 711-035-152, 1:5000). Membranes were developed using Luminata Crescendo Western HRP substrate (Millipore) and chemiluminescence was analyzed by exposure to Fuji Medical X-Ray Film (Fujifilm Europe GmbH).

### **In Vivo** T**ransduction of DRG Neurons With an Adenoviral Associated Virus Expressing hKAGd5-15**

The insert from pCAGGS-hKAGd5-15 plasmid (Table 1) was cloned into a control plasmid driven by cytomegalovirus (CMV) promoter (AAV1.CMV.PI.eGFP.WPRE.bGH) to obtain the hKAGd5-15 (eGFP-tagged, PKD-deletion-KIAA0319) plasmid (AAV1.CMV.PI.hKAd5-15-eGFP.WPRE.bGH). Control and hKAGd5-15-AAV vectors were produced by the Penn Vector Core at the University of Pennsylvania as described (Lock et al. 2010). Both mentioned plasmids were packaged in AAV2/1 particles (with AAV1 viral capsid and with AAV2 inverted terminal repeats). Genome copy (GC) titers of adeno-associated virus (AAV) vectors were determined by TaqMan (Applied Biosystems). Control AAV yielded 2.16 × 10^13^ GC/mL and hKAGd5-15 yielded 2.6 × 10^12^ GC/mL. For DRG injection, dorsal laminectomy was performed in 7-week old Wistar rats to expose L5 and L6 DRGs bilaterally (Yu et al. 2016) and 1 μL of AAV was injected in each DRG using a Hamilton syringe (33G) (*n =* 6 rats/group). Seven days after injection, bilateral sciatic nerve crush was performed and animals recovered for 3 days before sacrifice. Sciatic nerves were collected after 4% paraformaldehyde perfusion, cryoprotected in 30% sucrose and sectioned at 12 μm thickness. Image acquisition was performed using In Cell Analyzer 2000 and the length of regenerating eGFP-positive axons was measured from the tip of the axon to the lesion border using Fiji software. The length of regenerating axons (at least 20 axons per section) was quantified and at least 2 sections of each sciatic nerve were analyzed.

### 2C4 and Gamma-2-A (γ2A) Cell Lines

2C4 (parental control) and γ2A (that lack endogenous JAK2) cell lines are derived from human fibroblasts (Kohlhuber et al. 1997) and were a kind gift from Dr Ana P. Costa-Pereira (Imperial College, London). Both the parental cell line (2C4) and γ2A cells were cultured in DMEM supplemented with 10% FBS, 1% penicillin/streptomycin and 400 μg/mL G418.

### Downregulation of SH2B1, JAK2, and SMAD2 by shRNA in CAD Cells

CAD cells were transfected with shRNAs against either SH2B1 (shSH2B1#1: TRCN0000247807; shSH2B1#2: TRCN0000247811, Sigma), JAK2 (shJAK2#1: TRCN0000278125; shJAK2#2: TRCN0000023652; shJAK2#3: TRCN0000023649, Sigma), SMAD2 (shSMAD2#1: TRCN0000010477; shSMAD2#2: TRCN0000089336, Sigma) or empty vector (pLKO, Sigma), selected with 2 μg/mL puromycin for 48 h (a concentration and timepoint at which control nontransfected cells were not resistant to puromycin) and expanded. Puromycin-selected cell lines were then co-transfected with either full-length WT *KIAA*0319 or the corresponding empty vector and pmaxGFP™ (Lonza), as described above. Medium without serum was used 24 h post-transfection to promote differentiation and neurite outgrowth. Cells were fixed 96 h post-transfection and βIII-tubulin immunofluorescence was performed. Neurite length of eGFP-positive/βIII-tubulin-positive cells was measured using NeuronJ.

### Analysis of Axon Regeneration After Sciatic Nerve Injury

Sciatic nerve crush at the mid-thigh level was performed using Pean forceps, closed completely twice during 15s in *Thy1-cre*^*+*^*Kiaa0319*^*+/+*^ and *Thy1-cre*^*+*^*Kiaa0319*^*F/F*^ mice (*n =* 5 mice/group). Animals recovered for 3 days, after which collection and sectioning of sciatic nerves at 20 μm was performed. Consecutive longitudinal sections were collected for free-floating immunofluorescence with sheep anti-GAP-43 (1:5000; kindly provided by Dr Larry Benowitz, Harvard Medical School) and antigen detection was performed following incubation with biotinylated horse anti-goat (1:100; Vector) and streptavidin Alexa 568 (1:1000, Invitrogen). Image acquisition was done with Zeiss Axio Imager Z1 microscope (using the same settings for all the samples analyzed) and image analysis was performed with ImageJ. The lesion site was determined as the area with severely decreased YFP staining. GAP-43 staining intensity was determined distally from the proximal border of the injury site.

### Analysis of Axon Regeneration of Dorsal Column Fibers

Dorsal hemisection was performed, as described (Liz et al. 2014) using a micro ophthalmic scalpel (Feather, Safety Razor Co. Ltd) in *Thy1-cre*^*+*^*Kiaa0319*^*+/+*^ and *Thy1-cre*^*+*^*Kiaa0319*^*F/F*^ mice (*n =* 8 mice/group). Animals recovered for 5 weeks and 4 days prior to euthanasia, 2 μL of 1% cholera toxin-B (List Biologicals, Campbell, CA, USA) were injected in the left sciatic nerve. Serial spinal cord sagittal sections were collected for free floating immunohistochemistry with anti-cholera toxin-B (CT-B) (1:30 000; List Biologicals). Antigen detection was performed with biotinylated horse anti-goat (1:200; Vector) and streptavidin Alexa 568 (1:1000, Invitrogen). Image acquisition was done with a Laser Scanning Confocal Microscope (Leica SP5) and image analysis was performed with Fiji. Regeneration of dorsal column fibers was quantified by counting the total number of CT-B^+^/YFP^+^ axons within the glial scar. The length of the longest CT-B^+^/YFP^+^ axon found rostrally to the injury site was measured using as the origin a vertical line placed at the rostral end of the dorsal column tract.

### Statistics

Data are shown as mean ± SEM. For single comparisons, Student's *t*-test was used and for multiple comparisons, one-way ANOVA was chosen followed by Tukey's or Bonferroni's correction using Prism (GraphPad Software). When *P* < 0.05, differences were considered statistically significant.

## Results

### The Expression of KIAA0319 Increases With Age and is Downregulated After Injury

*Kiaa0319* expression in nervous tissue has been assigned to multiple regions in the developing mouse brain at sites where neuronal migration takes place, including the cortical plate of the neocortex (Paracchini et al. 2006). We examined the expression of this gene in other regions of the nervous system and determined that it is not restricted to the brain. Using in situ hybridization, we detected expression in spinal cord neurons (Fig. 1*B*) and sensory neurons within the DRG (Fig. 1*C*) of adult mouse (Fig. 1*B*,*C*, upper panels) and human (Fig. 1*B*, lower panels) tissues. RT-PCR was performed on RNA isolated from adult human DRG and spinal cord to further confirm *KIAA0319* expression (Fig. 1*D*). Interestingly, in mice, *Kiaa0319* expression increased from the early postnatal stage up to adulthood in the brain (Fig. 1*E*) and spinal cord (Fig. 1*F*). Moreover, this expression was strongly downregulated by injury as adult DRG neurons collected 24 h and 1 week after either sciatic nerve or spinal cord transection (Fig. 1*G*) showed significantly decreased levels of *Kiaa0319* mRNA (Fig. 1*H*). These data support KIAA0319 participation in additional functions unrelated to developmental neuronal migration and a possible KIAA0319-mediated repression of axon growth as its expression is increased in mature neurons and decreased upon injury to the nervous system.

### The Intracellular Domain of KIAA0319 is Required for Inhibition of Axon Growth

To test the hypothesis that KIAA0319 might be a negative regulator of axon growth, we overexpressed full-length human KIAA0319 (hKA) in neurons. KIAA0319 overexpression was confirmed by immunofluorescence in GFP-positive hippocampal neurons and CAD cells co-transfected with KIAA0319 and pmaxGFP™ (see Supplementary Fig. 1). In DRG neurons a tagged version of KIAA0319 was used (pCAGIG-hKA; Table 1). Overexpression of hKA in primary DRG neurons and in the neuronal cell line CAD significantly reduced total neurite length (Fig. 2*A*,*B*). We next tested the effect of KIAA0319 in primary hippocampal neurons, as this protein is expressed in the hippocampus (Peschansky et al. 2010) and as hippocampal neurons are highly polarized in culture with the axon being easily distinguished from the dendrites within 3–4 days in vitro (DIV) (Kaech and Banker 2006). Interestingly, overexpression of hKA led to a specific decrease in axon length without affecting dendrite length (Fig. 2*A*,*C*). Moreover, KIAA0319 overexpression significantly reduced branching not only in DRG neurons but also in hippocampal neurons and CAD cells (Fig. 2*D*). These data strongly support a KIAA0319-mediated repression of axon growth and branching in several different neuron types.

To assess which KIAA0319 domain is responsible for this effect, different KIAA0319 deletion mutants were generated (Fig. 2*E*; Table 1) and tested in primary hippocampal neuron cultures at DIV4 after transfection. Of note, expression of myc-His tagged versions of KIAA0319 did not interfere with its axon growth repressor activity (not shown). Overexpression of each KIAA0319 construct was validated by western blot analysis (Fig. 2*F*). When full-length hKA was overexpressed in CAD cells (Fig. 2*F*, hKA), several bands were detected under denaturing conditions, corresponding to partially (150 kDa) or fully (200 kDa) glycosylated forms, and to dimeric form(s) (~300 kDa), as described previously (Velayos-Baeza et al. 2008). KIAA0319 mutants lacking either the MANEC domain encoded by the first part of exon 3 (hKAd3a), the PKD domains encoded by exons 5–15 (hKAd5-15) or the complete extracellular portion of the protein (hKAd3-18) had a similar effect on axon growth as the full-length protein (Fig. 2*G*). These results suggest that at least in vitro, the extracellular domain of KIAA0319 is not required for repression of axon growth in hippocampal neurons.

In hippocampal neurons transfected with a mutant lacking the cytoplasmic domain of the protein (hKAd20-21), overexpression did not result in decreased axon length (Fig. 2*H*), suggesting that this domain is crucial for the activity of KIAA0319 in repressing axon growth. To specifically determine the region of the cytoplasmic domain of KIAA0319 responsible for this effect, we tested a mutant lacking the initial portion of the cytoplasmic domain (hKAd20-21a) and a mutant lacking the terminal portion of the cytoplasmic domain (hKAd21b). Whereas the hKAd21b mutant lacking the final portion of the cytoplasmic domain was still capable of inhibiting axon growth (Fig. 2*H*), the hKAd20-21a mutant lacking the initial portion of the cytoplasmic domain did not show such an effect (Fig. 2*H*). A deletion mutant lacking the highly conserved sequence SSLMVSE (Fig. 2*I*, dashed line on exon 21), present in both hKAd20-21a and hKAd21b deletion proteins, was still capable of inhibiting axon growth (Fig. 2*H*). These results strongly support that the initial region of the KIAA0319 cytoplasmic domain is necessary for axon growth inhibition. To further dissect this region, site directed mutagenesis of full-length hKA (in a pcDNA4 backbone, Table 1) was performed in several putative phosphorylation sites present in this sequence as predicted using PhosphoNET database (i.e., T993, Y995, Y1013, and S1019; Fig. 2*I*). Control hKA in pcDNA4 was overexpressed and the inhibition of axon growth was similar to that obtained with hKA in a pCAGIG backbone. For a matter of simplicity, in Fig. 2*H*, only the effect of hKA overexpression in a pCAGIG backbone is shown. Overexpression in hippocampal neurons of mutants in highly conserved putative phosphoresidues (Fig. 2*I*, arrows) revealed that hKA-Y995A was the only mutant capable of reverting the KIAA0319 effect as an axon growth repressor (Fig. 2*H*). In summary, our data show that the intracellular domain of KIAA0319 is responsible for its effect as a repressor of axon growth.

### KIAA0319 Modulates Axon Growth Through Smad2 Activation

**Figure 3.**
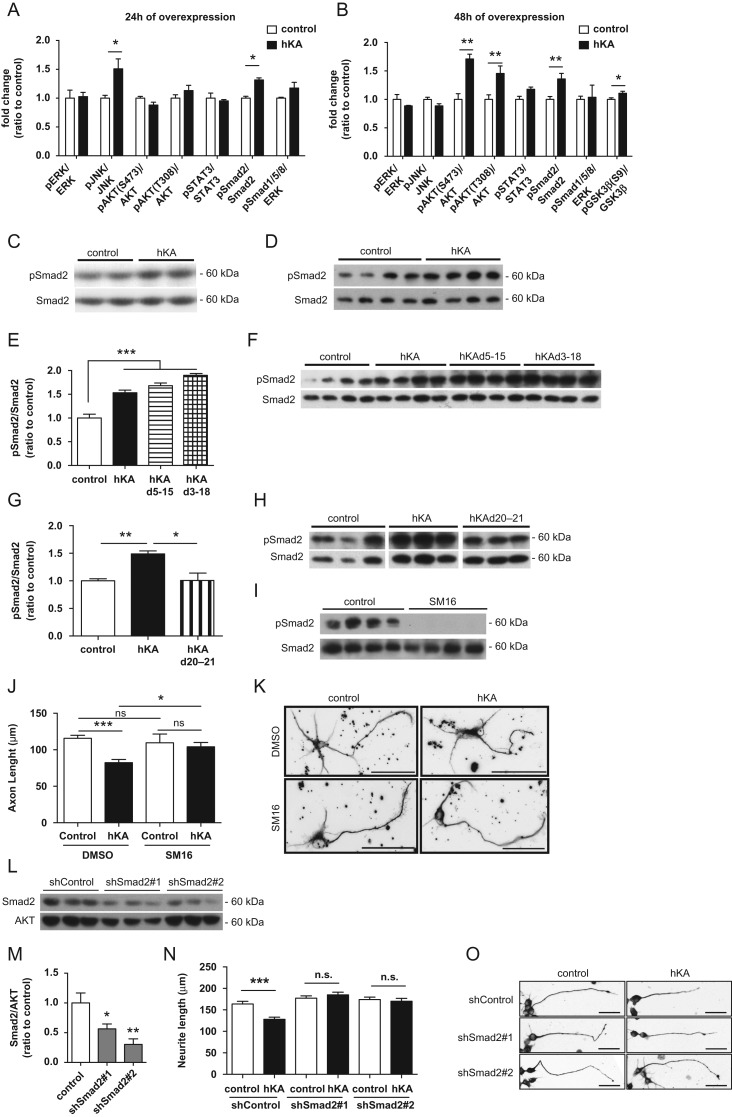
KIAA0319 inhibits axon growth through Smad2 activation. (*A, B*) Quantification of the ratios of phosphorylated/total ERK (pERK/ERK), JNK (pJNK/JNK), AKT (pAKT(S473)/AKT and pAKT(T308)/AKT), STAT3 (pSTAT3/STAT3), Smad2 (pSmad2/Smad2), Smad1/5/8 (Smad1/5/8/AKT), and GSK3β (pGSK3βS9/GSK3) as determined by western blot of CAD cell lysates collected either 24 h (*A*), or 48 h (*B*) after transfection with full-length WT human KIAA0319 (hKA); controls were CAD cells transfected with empty plasmid. (*C, D*) Representative anti-pSmad2 and anti-total Smad2 western blots 24 h (*C*) and 48 h (*D*) post-transfection of CAD cells with full-length WT human KIAA0319 (hKA). (*E–G*) Quantification of pSmad2 and Smad2 48 h post-transfection of CAD cells with either hKA or KIAA0319 mutants lacking the PKD domains (hKAd5-15) (*E*), the whole extracellular domain (hKAd3-18) (*F*), or a KIAA0319 mutant lacking the cytosolic domain (hKAd20-21) (*G*). (*F–H*) Representative western blots of *E* (*F*) and *G* (*H*). (*I*) Western blots of pSmad2 and total Smad2 in hippocampal neurons treated either with TGF-βRI inhibitor SM16 (SM16) or with vehicle DMSO (control). (*J*) Quantification of axon length in *I*; DMSO (control: *n =* 83; hKA: *n =* 64 neurons) or SM16 (control: *n =* 30; hKA: *n =* 44 neurons). (*K*) Representative images of βIII-tubulin immunofluorescence of GFP-positive hippocampal neurons transfected with either hKA or empty vector (control) and grown in the presence of SM16 or DMSO. Scale bar, 50 μm. (*L*) Western blot analysis of Smad2 levels in CAD cells transfected with shRNAs against Smad2 (shSmad2#1 and shSmad2#2) or a control plasmid (shControl). (*M*) Quantification of *L*. (*N*) Quantification of neurite length in CAD cells transfected with a control shRNA or specific shRNAs against Smad2 (shSmad2#1 and shSmad2#2) and overexpressing either KIAA0319 (hKA) or an empty vector (control); shControl (control: *n =* 143; hKA: *n =* 186 cells), shSmad2#1 (control: *n =* 155; hKA: *n =* 169 cells) and shSmad2#2 (control: *n =* 153; hKA: *n =* 104 cells). (*O*) Representative photomicrographs of βIII-tubulin immunofluorescence images of differentiated CAD cells transfected with either a control shRNA plasmid (shControl) and 2 shRNA against Smad2 (shSmad2#1 and shSmad2#2) and overexpressing either KIAA0319 (hKA) or an empty vector (control). Scale bar, 50 μm. Results are expressed in mean ± SEM. *P < 0.05, **P < 0.01, ***P < 0.001; ns, nonsignificant.

To unravel the molecular pathways triggered by KIAA0319, we analyzed the activation of key signaling molecules, including ERK, JNK, AKT, STATs, and Smads, 24 and 48 h after the overexpression of the full-length protein in CAD cells. Twenty-four hours after transfection of KIAA0319, phosphorylated levels of Smad2, and JNK were significantly increased (Fig. 3*A*,*C*) but only the increase of phosphorylated Smad2 was sustained 48 h after transfection (Fig. 3*B*,*D*). Interestingly, 48 h after transfection, phosphorylated AKT (S473 and T308) was also increased (Fig. 3*B*). Activated AKT mediates the downstream inactivation of GSK3β through phosphorylation of its S9 residue (Doble and Woodgett 2003). Accordingly, at this timepoint, phosphorylated GSK3β(S9) was slightly increased (Fig. 3*B*). The signaling pathway involving PI3K/AKT/GSK3β is important in the regulation of axon extension (Seira and Del Rio 2014). Recent work from our group using mouse models with different levels of GSK3β activity showed that reduced GSK3β activity results in increased axon growth (Liz et al. 2014). As such, GSK3β inactivation resulting from KIAA0319 overexpression is unlikely to be involved in the decrease of axon growth induced by this membrane protein. Given the early and sustained activation of Smad2 as a response to KIAA0319 overexpression, and its known role as a player in axon growth inhibition (Stegmuller et al. 2008; Hannila et al. 2013), we further dissected which KIAA0319 domain mediates this activation. Mutants lacking the extracellular domains of KIAA0319 (hKAd5-15 and hKAd3-18) were still capable of activating Smad2 (Fig. 3*E*,*F*). However, overexpression of the KIAA0319 mutant lacking the cytosolic domain (hKAd20-21) abolished the KIAA0319-mediated Smad2 activation (Fig. 3*G*,*H*). To determine whether Smad2 activation by KIAA0319 is necessary for KIAA0319-mediated inhibition of axon growth, we overexpressed KIAA0319 in hippocampal neurons treated with SM16, a small-molecule inhibitor of TGF-β type I receptor (TGF-βRI) that has high affinity for its ATP-binding site (Suzuki et al. 2007). SM16 blocks the phosphorylation of the glycine-serine rich domain of TGF-βRI and the downstream activation of Smad2 (Singh et al. 2003), with moderate off-target activity only against Raf and p38/SAPKa when tested against >60 related and unrelated kinases (Suzuki et al. 2007; Ling et al. 2008). 2 μM SM16, a concentration previously shown to almost totally prevent TGFβ-dependent elevation of phosphorylated Smad2 levels (Suzuki et al. 2007), was used. Of note, SM16 had no basal effect on axon growth in hippocampal neurons (Fig. 3*J*). SM16 effectively blocked Smad2 phosphorylation (Fig. 3*I*) and totally reverted the inhibitory effect of KIAA0319 on axon growth (Fig. 3*J*,*K*). To further reinforce Smad2 signaling as a key downstream target of KIAA0319, we downregulated Smad2 expression in CAD cells using 2 independent shRNAs (Fig. 3*L,M*). CAD cells with Smad2 downregulation and control CAD cells were subsequently transfected with full-length human KIAA0319 or control plasmid. Whereas decreased neurite outgrowth was observed in WT CAD cells upon KIAA0319 overexpression, no effect was observed in either Smad2-deficient cell line (Fig. 3*N,O*). Together, these results reinforce the role of the KIAA0319 cytosolic domain in the negative regulation of axon growth through Smad2 signaling.

### KIAA0319 Overexpression In Vivo Decreases Axon Regeneration

**Figure 4.**
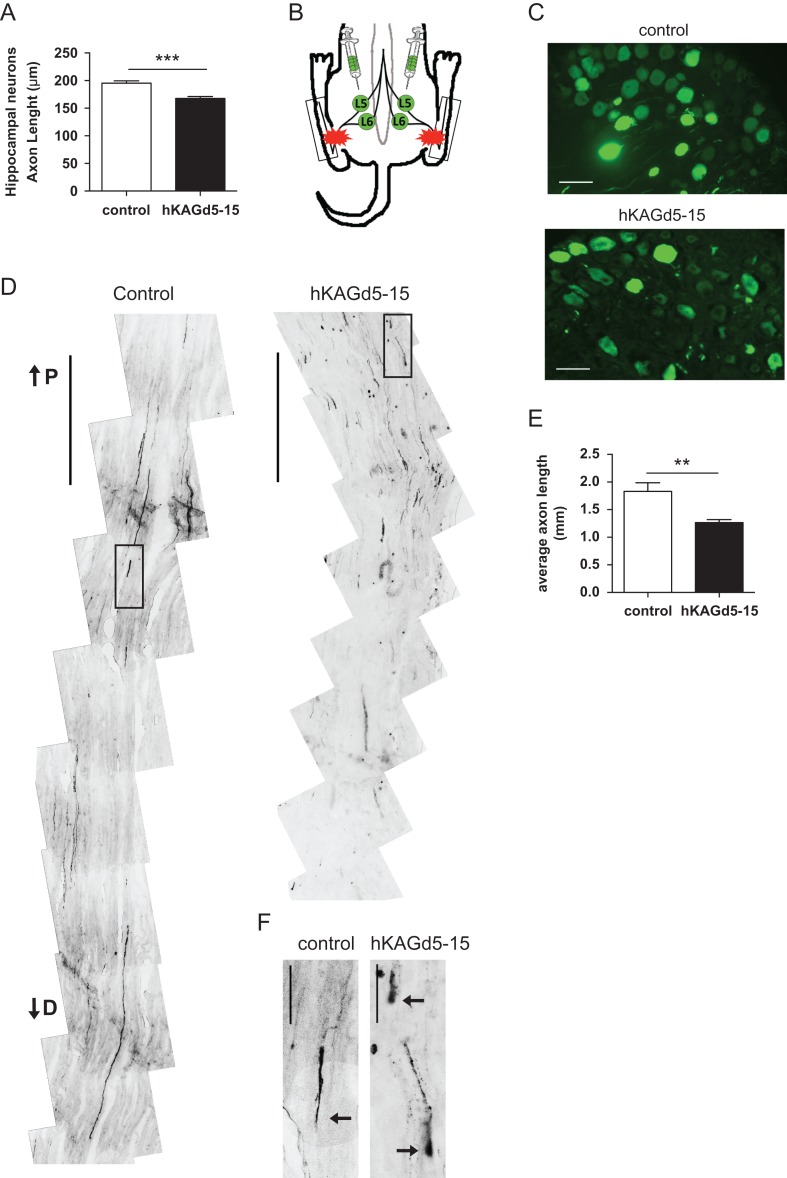
Overexpression of KIAA0319 decreases axon regeneration in vivo. (*A*) Quantification of axon length in hippocampal neurons transfected with control plasmid (control; *n =* 81 neurons) or hKAGd5-15 mutant (*n =* 76 neurons). (*B*) Schematic representation of AAV injections at L5 and L6 DRGs (green) and lesion site in the sciatic nerve (red stars). Boxes indicate the region of the sciatic nerve collected for analysis. (*C*) Representative micrographs of L5 DRGs 10 days after transduction with control AAV-eGFP or hKAGd5-15. Scale bar, 50 μm. (*D*) Representative micrographs of sciatic nerves from rats with DRGs transduced with either control eGFP (left) or hKAGd5-15 (right) expressing viruses. The micrographs presented were taken 300 μm distally of distance from lesion border. Scale bar, 500 μm. P, proximal; D, distal. (*E*) Quantification of the length of regenerating axons (considering as origin the distal border of the lesion site) of AAV-transduced DRGs with either control eGFP (*n =* 5 animals) or hKAGd5-15 (*n =* 6 animals). (*F*) Representative growth cones of control and retraction bulbs of hKAGd5-15 transduced DRGs. Axonal tips are highlighted with arrows. Scale bar, 50 μm. Results are expressed in mean ± SEM. **P < 0.01, ***P < 0.001.

Given the inhibitory role of KIAA0319 in axon growth in vitro, we tested the effect of its overexpression in vivo in the setting of sciatic nerve lesion, a paradigm that is followed by successful axon regeneration. We used AAV-based gene transfer in rat DRG neurons of a mutant form of eGFP-tagged KIAA0319 lacking the PKD domains (hKAGd5-15). This mutant was chosen due to AAV size constraints and because it had a similar effect to that displayed by full-length KIAA0319 both in terms of axon growth inhibition (Fig. 2*G*) and Smad2 activation (Fig. 3*E*). As a control, similar experiments were performed with a similar AAV virus carrying eGFP. Before in vivo delivery of the viruses, we evaluated the effect of the viral constructs in vitro*.* As expected, hippocampal neurons transfected with hKAGd5-15 plasmid had decreased axon length when compared with neurons transfected with the control construct (Fig. 4*A*). One week after viral injection in the DRG, bilateral sciatic nerve crush was performed (Fig. 4*B*) and axon regeneration was assessed by tracing of eGFP-positive axons distal to the crush. A robust eGFP expression was observed in DRGs transduced with either control or hKAGd5-15 viruses (Fig. 4*C*). Three days after injury, regenerating sensory axons injected with hKAGd5-15 had a 31% decreased axon length when compared with control eGFP-expressing axons (Fig. 4*D*,*E*). In contrast to the long axons with elongated growth cones observed in animals injected with control viruses (Fig. 4*D*,*F*), in hKAGd5-15 overexpressing neurons, regenerating axons accumulated closer to the lesion site and retraction bulbs were extensively observed (Fig. 4*D*,*F*). Together, these data strongly support that KIAA0319 is not only able to restrict axon growth in vitro but is also capable of inhibiting axon regeneration of DRG sensory neurons in vivo.

### The Intracellular Domain of KIAA0319 Engages JAK2 and SH2B1β to Induce Smad2 Activation and Decreased Axon Growth

**Figure 5.**
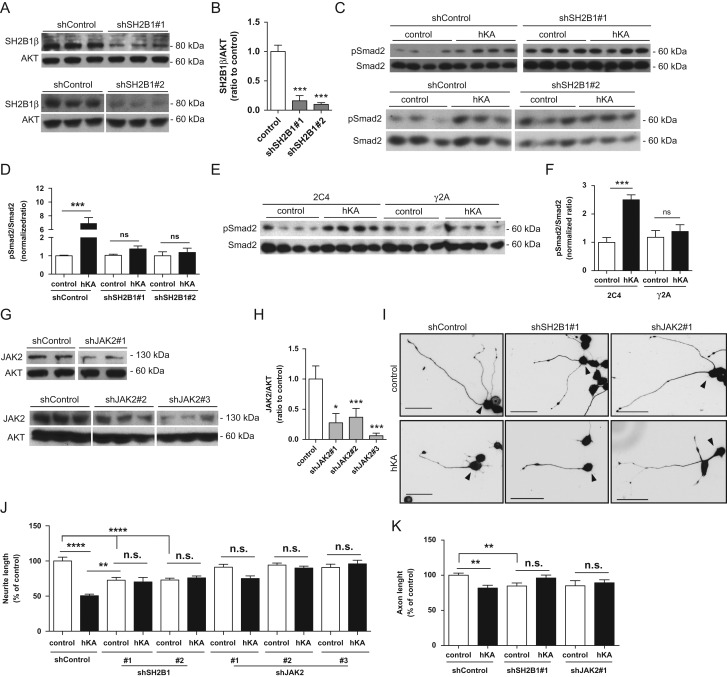
JAK2 and SH2B1β are necessary for KIAA0319-induced activation of Smad2 and inhibition of axon growth. (*A*) Western blot analysis of SH2B1β levels in CAD cells transfected with different shRNA against SH2B1 (shSH2B1#1, upper panel; and shSH2B1#2, lower panel) or a control plasmid (shControl) following puromycin selection. (*B*) Quantification of *A*. (*C*) Western blot analysis of pSmad2 and total Smad2 levels in CAD cells depleted of SH2B1 (shSH2B1#1, upper panel and shSH2B1#2, lower panel) or transfected with a control shRNA plasmid (shControl) overexpressing either KIAA0319 (hKA) or an empty vector (control). (*D*) Quantification of *C*. (*E*) Western blot analysis of pSmad2 and Smad2 levels in JAK2-deficient cells (gamma-2-A; γ2A) and in the control parental cell line (2C4) upon overexpression of KIAA0319 (hKA) or an empty vector (control). (*F*) Quantification of *E*. (*G*) Western blot analysis of JAK2 levels in CAD cells transfected with different shRNA against JAK2 (shJAK2#1, upper panel; shJAK2#2 and shJAK2#3, lower panel) or a control shRNA, following puromycin selection. (*H*) Quantification of *G*. (*I*) Representative photomicrographs of anti-βIII-tubulin immunofluorescence in differentiated GFP-positive CAD cells (highlighted with an arrowhed) stably transfected with either a control shRNA plasmid (shControl), a shRNA against SH2B1 (shSH2B1#1), or a shRNA against JAK2 (shJAK2#1) and overexpressing either KIAA0319 (hKA) or an empty vector (control). Scale bar, 50 μm. (*J*) Quantification of total neurite length of *I*; shControl (control: *n =* 162; hKA: *n =* 152 cells), shSH2B1#1 (control: *n =* 112; hKA: *n =* 72 cells), shSH2B1#2 (control: *n =* 201; hKA: *n =* 149 cells), shJAK2#1 (control: *n =* 126; hKA: *n =* 89 cells), shJAK2#2 (control: *n =* 136; hKA: *n =* 167 cells) and shJAK2#3 (control: *n =* 80; hKA: *n =* 87 cells). (*K*) Quantification of axon length of hippocampal neurons transfected with both hKA and different shRNAs- shControl (control: *n =* 188; hKA: *n =* 67 cells), a shRNA against SH2B1 (shSH2B1#1; control: *n =* 78; hKA: *n =* 56 cells) or a shRNA against JAK2 (shJAK2#1; control: *n =* 32; hKA: *n =* 81 cells). Results are expressed in mean ± SEM. **P < 0.01, ***P < 0.001, ****P < 0.0001; ns = not statistically significant.

The pathway triggered by KIAA0319 is presumably initiated by interaction with its signaling partners. The cytoplasmic domain of KIAA0319 was suggested to interact with SH2B1β signaling protein, as determined by yeast-two-hybrid (Nakayama et al. 2002). SH2B1 belongs to the SH2B family and is an adaptor protein with 4 splicing variants (α, β, γ, δ) with distinct C-terminal sequences (Yousaf et al. 2001). SH2B1 is ubiquitous, interacting with multiple receptor tyrosine kinases (Maures et al. 2007). To assess if SH2B1 regulates KIAA0319 function, we knocked-down SH2B1 expression in CAD cells using specific shRNAs (Fig. 5*A*,*B*). SH2B1 depleted CAD cells and control neurons were subsequently transfected with full-length human KIAA0319 or a control plasmid, and Smad2 phosphorylation was assessed by western blot. Unlike control cells expressing SH2B1, which induced Smad2 phosphorylation upon overexpression of KIAA0319, cells depleted of SH2B1 failed to induce Smad2 phosphorylation (Fig. 5*C*,*D*). These data suggest that SH2B1 is necessary for the activation of Smad2 induced by KIAA0319.

SH2B1β has been extensively described as a Janus Kinase 2 (JAK2) substrate. JAK2 binds to SH2B1β via its SH2-domain promoting tyrosyl phosphorylation of SH2B1β which, in turn, increases JAK2 activation (Rui and Carter-Su 1999). To check whether JAK2 is also a partner of KIAA0319 signaling, we overexpressed KIAA0319 in gamma-2-A (γ2A) cells, a cell line derived from human fibroblasts specifically depleted of JAK2 (Kohlhuber et al. 1997) and in the parental WT cell line 2C4. Interestingly, whereas KIAA0319-dependent Smad2 activation occurred in the parental cell line, γ2A cells overexpressing KIAA0319 failed to induce Smad2 activation (Fig. 5*E,F*). These results suggest that JAK2 is a crucial player in KIAA0319 intracellular signaling.

To determine whether defective Smad2 activation by KIAA0319 in the absence of SH2B1 and JAK2 correlates with reversion of axon growth inhibition, we evaluated neurite outgrowth in KIAA0319-overexpressing CAD cells depleted of either SH2B1 (Fig. 5*A*,*B*) or JAK2 (Fig. 5*G*,*H*). Whereas total neurite length was severely diminished in WT CAD cells by KIAA0319 overexpression, this KIAA0319-mediated effect was not observed in CAD cells in which either SH2B1 or JAK2 were depleted (Fig. 5*I*,*J*). Of note, as previously reported (Maures et al. 2009; Shih et al. 2013), the knockdown of SH2B1 restricted neurite outgrowth (however to a lower extent than KIAA0319 overexpression) (Fig. 5*J*) but this reduction was not further exacerbated by the presence of KIAA0319. Similarly, in primary hippocampal neurons, overexpression of KIAA0319 and downregulation of SH2B1 (see Supplementary Fig. 2) or JAK2 (see Supplementary Fig. 3) also reverted the inhibitory effect of KIAA0319 in axon growth (Fig. 5*K*), further supporting the physiological involvement of JAK2/SHB21 in KIAA0319-mediated repression of axon growth. In summary, our results support that KIAA0319 is a negative regulator of axon growth that engages the JAK2-SH2B1 pathway through its cytosolic tail to activate Smad2 and decrease neurite outgrowth.

### **KIAA0319 Deletion Results in Increased Axon Growth In Vitro and Increased Axon Regeneration** **In Vivo**

**Figure 6.**
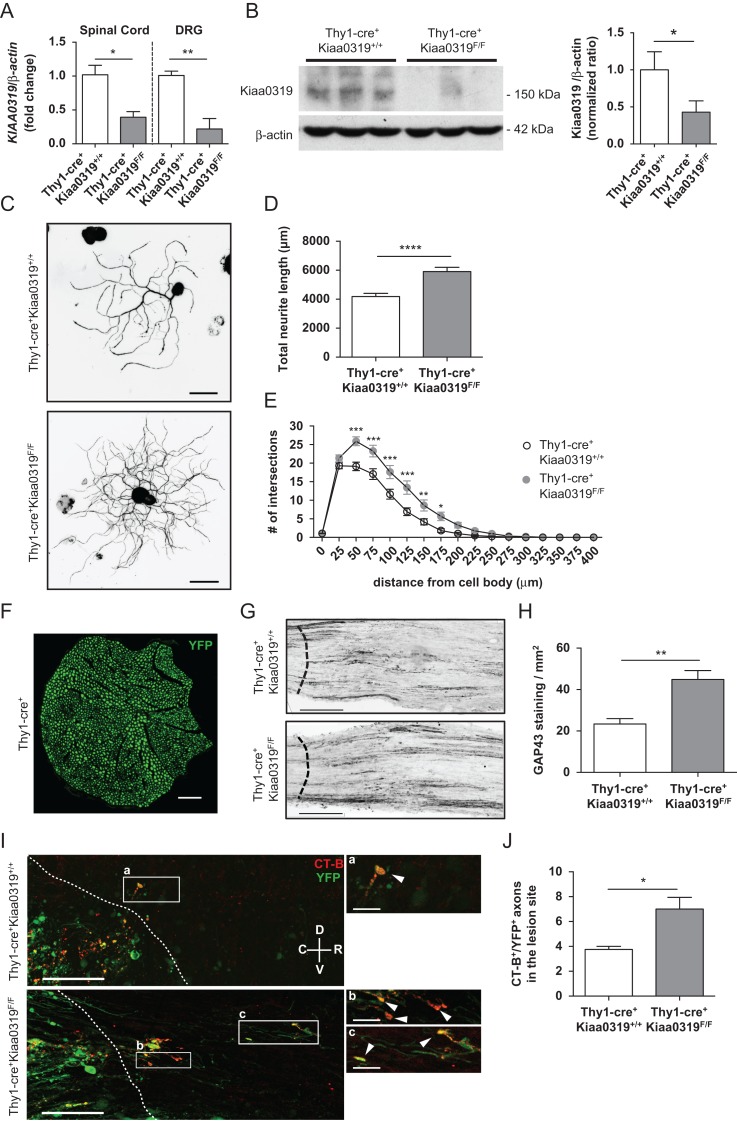
Deletion of Kiaa0319 increases axon growth. (*A*) qPCR analysis of *Kiaa0319* in the spinal cord and DRG of *Thy1-cre*^*+*^*Kiaa0319*^*+/+*^ and *Thy1-cre*^*+*^*Kiaa0319*^*F/F*^ animals. (*B*) Western blot analysis of KIAA0319 (left) and respective quantification (right) in spinal cord lysates of *Thy1-cre*^*+*^*Kiaa0319*^*+/+*^ and *Thy1-cre*^*+*^*Kiaa0319*^*F/F*^ animals. (*C*) Representative photomicrographs of anti-βIII-tubulin staining of YFP-positive DRG neurons isolated from adult *Thy1-cre*^*+*^*Kiaa0319*^*+/+*^ and *Thy1-cre*^*+*^*Kiaa0319*^*F/F*^ mice plated on laminin and grown for 12 h. Scale bar, 50 μm. (*D*) Quantification of the total neurite length of *C*. (*E*) Sholl analysis of DRG neurons from *Thy1-cre*^*+*^*Kiaa0319*^*+/+*^ and *Thy1-cre*^*+*^*Kiaa0319*^*F/F*^ animals. (*F*) Representative photomicrographs of *Thy1-Cre*^*+*^ mice sciatic nerve in transverse section. Scale bar, 500 μm. (*G*) GAP43 staining in sciatic nerves of *Thy1-cre*^*+*^*Kiaa0319*^*+/+*^ and *Thy1-cre*^*+*^*Kiaa0319*^*F/F*^ mice isolated 3 days after sciatic nerve crush. The dashed line indicates the lesion border. Scale bar, 200 μm. (*H*) Quantification of *F* (*n =* 5 each genotype). (*I*) Representative photomicrographs of CT-B staining in spinal cords of *Thy1-cre*^*+*^*Kiaa0319*^*+/+*^ and *Thy1-cre*^*+*^*Kiaa0319*^*F/F*^ mice after dorsal column hemisection. The dashed line indicates the lesion border; C, caudal; R, rostral; V, ventral; D, dorsal. Scale bar, 100 μm. A higher magnification of the boxed regions (*a–c*) is shown in the right panels. Arrowheads highlight CT-B^+^ (red)/ YFP^+^ (green) axons. Scale bar, 25 μm. (*J*) Quantification of the average number of axons within the glial scar in *Thy1-cre*^*+*^*Kiaa0319*^*+/+*^ and *Thy1-cre*^*+*^*Kiaa0319*^*F/F*^ mice (*n =* 8 each genotype). Results are expressed in mean ± SEM. *P < 0.05, **P < 0.01, ***P < 0.001, ****P < 0.0001.

As our data strongly support that KIAA0319 is a repressor of axon growth, we generated a mouse model with an inducible *Kiaa0319* specific deletion in neurons to assess whether the absence of Kiaa0319 results in increased axon regeneration. For that we crossed *Kiaa0319*^*F/F*^ mice with Slick-H mice that co-express both inducible-CreERT2 and YFP under the control of the neuronal *Thy1* promoter (Young et al. 2008). qRT-PCR using a primer in the targeted exon 6 validated the decreased *Kiaa0319* expression in the spinal cord and DRG (60% and 80% reduction, respectively) of *Thy1-cre*^*+*^*Kiaa0319*^*F/F*^ mice when compared with *Thy1-cre*^*+*^*Kiaa0319*^*+/+*^ (Fig. 6*A*). Western blot analysis of the spinal cord was performed to confirm the deletion of KIAA0319 at the protein level. A 58% decrease was observed in *Thy1-cre*^*+*^*Kiaa0319*^*F/F*^ when compared with control *Thy1-cre*^*+*^*Kiaa0319*^*+/+*^ mice (Fig. 6*B*) which correlates well with the qPCR data (Fig. 6*A*). Supporting our previous findings, *Thy1-cre*^*+*^*Kiaa0319*^*F/F*^ DRG neurons had an increased total neurite length (Fig. 6*C*,*D*) and branching (Fig. 6*E*) when compared with control *Thy1-cre*^*+*^*Kiaa0319*^*+/+*^ neurons. To determine whether increased neurite outgrowth in vitro in the absence of KIAA0319 was related to increased axon regeneration in vivo, *Thy1-cre*^*+*^*Kiaa0319*^*+/+*^ and *Thy1-cre*^*+*^*Kiaa0319*^*F/F*^ mice were subjected to sciatic nerve crush and axon regrowth was assessed using GAP-43 staining (Woolf et al. 1990). Of note, in the *Thy1-cre*^*+*^ line, all the myelinated axons in the sciatic nerve are YFP-positive (Fig. 6*F*). 3 days after sciatic nerve crush, GAP-43 immunostaining was increased in *Thy1-cre*^*+*^*Kiaa0319*^*F/F*^ animals when compared with controls (Fig. 6*G*,*H*) supporting a higher regenerative capacity in the absence of Kiaa0319. To determine if a similar response was obtained after spinal cord injury, dorsal column hemisection was performed and axon regeneration of ascending dorsal column sensory fibers was assessed after cholera toxin B injection in the sciatic nerve. *Thy1-cre*^*+*^*Kiaa0319*^*F/F*^ animals had an increased number of YFP-positive axons with the ability to grow within the glial scar (Fig. 6*I*,*J*) and only axons from *Thy1-cre*^*+*^*Kiaa0319*^*F/F*^ were capable of regrowing for distances longer than 100 μm (Fig. 6*I*, box c). These results support the absence of KIAA0319 as increasing the intrinsic regenerative capacity of axons and reinforce the hypothesis that KIAA0319 plays an inhibitory role in axon growth and regeneration.

## Discussion

Our work establishes the dyslexia-associated TM neuronal protein KIAA0319 as an inhibitor of axon growth, both in embryonic and adult neurons. This effect is released in the adult injured nervous system, as *KIAA0319* expression is downregulated. During neuronal development, the growth cones at the tip of the projected axons are continuously sensing the surrounding environment, navigating through the recognition of attractive and repulsive cues. Among these guidance cues, Robo1 is an important player (Kidd et al. 1998). In dyslexic individuals genetically linked to a specific haplotype of *ROBO1*, the expression of this gene is decreased (Hannula-Jouppi et al. 2005) similarly to what is described for *KIAA0319* (Paracchini et al. 2006). Interestingly, and again similarly to *KIAA0319*, overexpression of Robo1 decreases axon growth whereas silencing of Robo1 or of its ligand, Slit1, promotes neurite extension (Mire et al. 2012). Together these findings suggest that in dyslexia, the dysregulation of pathways that negatively regulate axon growth is of high relevance.

Here we show that KIAA0319 inhibitory activity is dependent on JAK2-SH2B1β signaling and Smad2 activation. Smad2 activation has been previously identified as a key signaling pathway inhibiting axon growth. During development, phosphorylated Smad2 interacts with SnoN leading to impaired neuritogenesis, and the knockdown of Smad2 enhances neurite outgrowth in the presence of inhibitory substrates such as myelin (Stegmuller et al. 2008). Later, Smad2 was further demonstrated to be an axon growth inhibitor that participates in myelin-mediated inhibition (Hannila et al. 2013). Of note, inactivation of the Robo1 pathway also decreases Smad2 activation (Chang et al. 2015). Interestingly, activation of Smad signaling through the bone morphogenetic (BMP) pathway, which leads to the phosphorylation of Smad1, 5, and 8, has the opposite effect, resulting in enhanced axon growth (Parikh et al. 2011). The fate of a growing/regenerating axon may therefore be highly dependent on competing Smad transcriptional pathways.

Apart from Smad2 activation, KIAA0319 leads to increased AKT phosphorylation, which is a known effect of SH2B1β activation (Wang et al. 2004; Lu et al. 2010). SH2B1β is an SH2 domain-containing adaptor protein that can be recruited and phosphorylated by multiple ligand-activated receptor tyrosine kinases and cytokine receptor-associated JAK family kinases. Of note, SH2B1β binds the tyrosine kinase A (TrkA) receptor in response to NGF binding (Rui et al. 1999) to promote NGF-related gene transcription and neurite outgrowth (Maures et al. 2009). However, SH2B1β is required for KIAA0319-mediated axon growth inhibition. How SH2B1β exerts opposing effects when it is downstream of different receptors is unknown but one possibility might be that KIAA0319 competes with TrkA for SH2B1-mediated signaling. In this respect, the SH2B family member SH2B3 negatively modulates axon growth in PC12 cells through a mechanism involving the competition of TrkA binding with the positive-acting SH2B1β (Wang et al. 2011). SH2B1β binds and potentiates JAK2 phosphorylation (Rui and Carter-Su 1999). JAK2 is a key signaling protein activated by several receptors including the receptors for erythropoietin (Witthuhn et al. 1993), growth hormone, and leptin (Maures et al. 2007). JAK2 can be activated in response to TGF-β stimulation (Dees et al. 2012), which supports, similarly to what occurs following KIAA0319 overexpression, a link between JAK2 and Smad2 signaling. Also in support of a link between the SH2B1β-JAK2 pathway and Smad2 activation, SH2B1β enhances JAK2 activity and binds directly to filamin A (Rider and Diakonova 2011) which may function as a cytoplasmic anchor for Smad2 (Sasaki et al. 2001).

In vitro, our results show that the cytosolic KIAA0319 tail, specifically the juxtamembrane region of the cytoplasmic domain, is sufficient for Smad2 activation and axon growth inhibition. The Tyr995 residue, which lies in this domain, is here shown to be an important residue for activity of KIAA0319. Of note, the KIAA0319 Y995A mutant impairs KIAA0319 internalization from the plasma membrane (Levecque et al. 2009), which may be the reason behind the observed effect instead of a possible alteration of the phosphorylation status of the protein. Our results also support that the activity of KIAA0319 is independent of its extracellular domain. Examples of receptors that are constitutively active in the absence of their extracellular domains include the thyrotropin (TSH) receptor (Zhang et al. 1995) and epidermal growth factor (EGF) receptor (Voldborg et al. 1997). In this respect, one should consider that the KIAA0319 homologous protein, KIAA0319-like, that has also been associated with developmental dyslexia (Couto et al. 2008), has been reported as an interactor of Nogo Receptor 1 (NgR1) (Poon et al. 2011). NgR1 is a glycosyl phosphatidylinositol (GPI)-anchor protein that binds to Nogo-66 (Fournier et al. 2001), the inhibitory portion common to all 3 isoforms of Nogo (A, B, and C), to promote growth cone collapse and inhibit neurite outgrowth in several neuronal types (GrandPre et al. 2000). Given the GPI nature of NgR1, it lacks an intracellular domain thereby requiring additional neuronal TM proteins to transduce the inhibitory signals (Schwab and Strittmatter 2014). NgR1 might therefore interact with KIAA0319, leading to activation of the JAK2-SH2B1β signaling cascade, restricting elongation, although this hypothesis is yet to be explored. To date, this is the first work that starts to unravel the signaling pathway triggered by KIAA0319 in neurons and that establishes this dyslexia-associated TM neuronal protein as a player in axon growth and regeneration.

## Supplementary Material

Supplementary Data
